# Striving to be prepared for the painful: Management strategies following a family member's diagnosis of advanced cancer

**DOI:** 10.1186/1472-6955-10-18

**Published:** 2011-10-04

**Authors:** Catarina Sjolander, Berith Hedberg, Gerd Ahlstrom

**Affiliations:** 1 The School of Health Sciences, Jönköping University, Jönköping, Sweden; 2 The Ryhov County Hospital, Jönköping, Sweden; 3 The Swedish Institute for Health Sciences, Lund University, Lund, Sweden

## Abstract

**Background:**

Cancer has consequences not only for the sick person but also for those who have a close relationship with that person. Greater knowledge about how family members manage the situation in the period immediately following the diagnosis means greater opportunity to provide the best possible support for the family. The purpose of this study was to explore management strategies that family members use when the patient is in the early stage of treatment for advanced cancer.

**Methods:**

Twenty family members of cancer patients were included in the study shortly after the diagnosis. The patients had been diagnosed 8-14 weeks earlier with advanced lung cancer or gastrointestinal cancer. The data were collected in interviews with family members and subjected to qualitative latent content analysis. Through the identification of similarities and dissimilarities in the units of meaning, abstraction into codes and sub-themes became possible. The sub-themes were then brought together in one overarching theme.

**Results:**

The overall function of management strategies is expressed in the theme *Striving to be prepared for the painful*. The family members prepare themselves mentally for the anticipated tragedy. Family relationships become increasingly important, and family members want to spend all their time together. They try to banish thoughts of the impending death and want to live as normal a life as possible. It becomes important to family members to live in the present and save their energy for the time when they will need it the most. How participants handle their worries, anxiety and sadness can be categorized into seven sub-themes or management strategies: Making things easier in everyday life, Banishing thoughts about the approaching loss, Living in the present, Adjusting to the sick person's situation, Distracting oneself by being with others, Shielding the family from grief, and Attempting to maintain hope.

**Conclusions:**

The findings revealed that the family members have their own resources for handling the early stage of the cancer trajectory in an acceptable way. There is a need for longitudinal studies to generate knowledge for designing evidence-based intervention programmes that can prevent future ill-health in these vulnerable family members.

## Background

When a member of the family has been diagnosed with advanced cancer, this has a great impact not only on that family member, but on the rest of the family too. Confronted with the life-threatening situation of their loved one, family members experience shock, psychological distress and a sense of vulnerability [1-3]. There are often symptoms of anxiety, sadness and depression in family members of persons with cancer [4,5].

The increased use of outpatient services for cancer treatment, with less time in the hospital, creates an increased need for better understanding of the family members' role as caregiver to a person with advanced cancer [6-8]. Family members are often overwhelmed by the care burden with its emotional, social and financial demands [1]. It often causes them to neglect their own needs, both physical and emotional [9]. The psychological and physical stress exposes family members, in particular those who are caregivers, to greater risk of ill-health [9].

In the literature, there is an interest in understanding factors that can prevent distress and ill-health in family members taking care of a significant other who has advanced cancer. Of importance with regard to the family members' well-being are strategies for managing stressful issues in everyday life [10-12]. In the existing literature, management of distress is typically defined as coping. An immense amount of research has been based on Lazarus and Folkman's cognitive theory [13-15]. This theory focuses on what a person actually thinks, feels and does in a specific encounter. Coping is, from this perspective, contextual and influenced by the person's appraisal of the particular demands and available resources for managing them. Most of the studies on family members of a person with cancer have focused on cases of breast or prostate cancer, while the consequences of cancers with a worse prognosis have been less frequently studied [16]. Among the four most common cancers that cause death in Europe are lung cancer and stomach cancer [17]. Health-care professionals encountering family members of persons with cancer who have a poor prognosis need knowledge about how well the family members can manage the situation they face. Provision of high-quality cancer care services should include strategic support that takes into account the family members' own resources. Greater knowledge about how family members manage shortly after the diagnosis means greater opportunity to provide the best possible support for the family. The purpose of the present study was to explore management strategies that family members employ when the patient is in the early stage of treatment for advanced lung or gastrointestinal cancer.

## Methods

### Design and setting

A qualitative approach was used, with interviews designed to acquire deeper knowledge about family members' management strategies. Ethical approval for this study was granted by the Research Ethics Committee at Linköping University, Sweden. Informed consent was obtained from all participants prior to the study. It was made clear that participation was voluntary and that they were free to withdraw from participation whenever they wished. Confidentiality was guaranteed, and the findings could not be linked to individuals.

### Participants

Each participant was a family member of a patient who 8-14 weeks earlier had been diagnosed with advanced lung cancer (n = 10) or gastrointestinal (pancreatic, oesophageal, liver, biliary or stomach) cancer (n = 10). To obtain enough variations in data, the inclusion criteria stipulated that half of the family members represent persons with lung cancer and half represent those with gastrointestinal cancer. Twenty patients (13 men and 7 women; median age 72 years) selected one family member to participate. At the time of data collection, all 20 patients were having chemotherapy treatment, two were having radiation treatment, and one had undergone and one was in preparation for surgical therapy.

Nurses and physicians at three hospitals in the south of Sweden asked persons with cancer who had a family member if they were willing for this person to have written information about participation in the study. The patients received two letters indicating the purpose and design of the study, one for themselves and one to give to a family member. Patients were instructed to choose a family member they wanted to ask to participate.

All of the 20 family members gave their written consent to participate and were contacted by the interviewer to arrange a time and place for the interview. The study included 16 women and 4 men (age range 31-77 years, median age 60). Background characteristics are shown in Table 1.

**Table 1:** Background characteristics of the family members

	All (n = 20)
	n (%)
**Living situation**	
Sharing household with the person with cancer	13 (65)
Separate household	7 (35)
	
Children or teenagers at home	7 (35)
Grown children and grandchildren not sharing household	13 (65)
	
**Relationship to the person with cancer**	
Partner (eight wives, three husbands)	11 (55)
Cohabitant	2 (10)
Grown child	5 (25)
Other relative (one ex-partner, one uncle)	2 (10)
	
**Work status**	
Currently working	13 (65)
Retired	5 (25)
Student	1 (5)
On sick leave from work	1 (5)

### Interview procedure

Eleven of the interviews were conducted at the hospitals, seven in the family members' homes and two at their places of work. The interviews were each conducted by the first author (CS) on a single occasion, tape-recorded and transcribed verbatim. Two open questions were asked regarding how the family members handled their everyday lives: (1) How does your close relative's cancer affect your day-to-day life? (2) How do you manage the situation? Follow-up questions were asked to clarify and add more details to the narrative answers given (e.g. What do you *think *about the situation? How do you *feel *about the situation? What do you *do *about the situation? Have you anything more to add about that?) The follow-up questions were based on aspects in the coping literature [13-15], and the number of these questions depended on the richness of the participant's answer to the first two questions. The duration of the interview was 60-90 minutes (75 minutes on average).

### Data analysis

The method used was based on latent content analysis for narrative text [18-20]. Content analysis was chosen because it is an inductive process, involving openness to whatever the participants said, though within the limitations of the study's purpose of exploring management strategies. First, the interview text was read through several times, as open-mindedly as possible, in an attempt to grasp its overall meaning. It was then divided into units of meaning. These units were first condensed on a descriptive level (keeping close to the original text) and then abstracted into codes, which involved interpretation of the underlying meaning. Through identification of similarities and dissimilarities in the units of meaning and codes, further abstraction could be achieved, from which sub-themes emerged. Finally, the sub-themes were brought together under a single overarching theme. The results of the analysis, at each step, were discussed by all three authors, and the refined codes, sub-themes and themes constituted the findings [20].

## Results

The main findings illustrated the function of the theme of management: *Striving to be prepared for the painful*. This theme was based on seven sub-themes or management strategies: (1) Making things easier in everyday life, (2) Banishing thoughts about the approaching loss, (3) Living in the present, (4) Adjusting to the sick person's situation, (5) Distracting oneself by being with others, (6) Shielding the family from grief and (7) Attempting to maintain hope.

### Striving to be prepared for the painful

The family members are filled with the sense of a tragic future, knowing in their hearts that a great loss is approaching. Yet at the same time, they try to banish this kind of thinking and live as normal a life as possible. They want to maintain the life they are used to, life as it was before the onset of the disease. The family members try to make things easier by making practical changes for themselves and their families in everyday life. It becomes important to live in the present and embrace the happiness to be found in the sick person's still being alive. The family members adapt to the person's situation and want to be there for their loved one as much as they can. They see a meaning in their role as family member, and there is nothing they want more than to lighten the other's burden. Family life becomes increasingly important, and family members spend all the time they can together. To have enough energy to manage the new situation, the family members need the distraction of being with others and thinking of something else for a short while before coming back to the family. They draw strength from the sense of family community, strength that helps them bear the burden and be supportive within the family. Up until the sick person's death, the family members shoulder the grief of the rest of the family and spare them emotional strain. As long as the person is still alive, there is hope of improvement. By living in the present, it is possible to find escape from thoughts about the menacing future. This future, after all, is not here *now*, so there is hope. There is a belief in the chance that the sick person will live a while longer, even a belief in the possibility of a miracle occurring.

#### 1. Making things easier in everyday life

The family members make practical changes for themselves and their families in everyday life. They try to reduce their work hours and plan how to make things easier in the changed family situation, including preparing for the need to take time off when the pressure becomes too great. The family members also make arrangements to prepare for increased caregiving for the sick person at home. At the same time, they feel vulnerable with new demands in everyday life. Whilst problems can be tackled as they occur, thoughts about the menacing future, about death and about being left alone, are never far away. There is a deep fear of loneliness and the loss of their relationship with the sick person. These overwhelming feelings cause them to seek emotional support and understanding from others, particularly from those who have experienced crises of their own. This emotional relief makes everyday life easier to live. When they can show their feelings to their friends and co-workers, the family members gain an understanding of their situation. Those who are parents with one or more small children want to be able to cope on their own when the sick person no longer has energy to share the practical things in everyday life:

I've begun to realize that I must take hold of my life now because I need to manage myself with the kids. I worked in a shop before. I've done it for many years, but it is inconvenient because of the hours. It is only open until six, but it's still too long for the children not to be cared for properly. My husband cannot take care of the children. He is too tired. Now, I am studying so it's going to be a change in future. I'll try to get better working hours. (Female, wife, age 35)

#### 2. Banishing thoughts about the approaching loss

It is painful and stressful to be aware of the sick person's incurable cancer and probable death. The family members do not know how long the person is going to live, nor do they really *want *to know. They are afraid of the menacing future and try to banish all thoughts of it. It is stressful for them to think about the disease. The family members oscillate between an awareness of the limited life-expectancy of the sick person and an effort to banish the thought of loss. To cope with the situation and be able to function as the persons they are, they suppress or distance themselves from thoughts about the future. They distance themselves emotionally in the face of the threatened loss of the other and try to live in their customary way in order to cope with the demands of everyday life. The anxiety of knowing that the sick person is probably going to die is countered by an attempt to think positive thoughts (such as that the person may have a few years left):

*When we first found out about it, I went down to half-speed. It came as such a shock. But I suppose it's become a bit easier to keep it at a bit of a distance, so as to be able to carry on. *(Female, daughter, age 48)

#### 3. Living in the present

The family members feel an uncertainty about the future with its threat of the loss of the sick person. They cannot make plans for a year ahead, or indeed even a month, because the gravity of the prognosis means that there is no knowing whether they can keep to a plan. The sick person has undergone the first treatment, and the initial shock has been dampened. There is a changed conception of the present and a changed conception of the possibility of planning for the future. The family members concentrate their energy on the here and now, taking each day as it comes. Things are as they are, but this helps.

Thoughts about the menacing future are still there. Nevertheless, the family members can find a certain peace in the present (while life-sustaining treatment is being given). They remind themselves that it is important that they live in the present and take advantage of the joy life offers. Their perspective has changed from being future-oriented to being directed towards the present, and the family members perceive it as important to fully participate in life as it is here and now:

*The illness hasn't taken over our lives because he's so strong that we can live together, which means a lot. I don't know what it'll be like at the end when he leaves me. So I say to myself: 'Why go through that grief now?' I mean, it's better to concentrate on the happiness we've got today. *(Female, wife, age 77)

#### 4. Adjusting to the sick person's situation

The family member's daily plans depend to a great extent on how the person with cancer is feeling. They consider their support and role as a family member meaningful, and they want to lighten the sick person's burden. Everyday life revolves around the illness, and they want to be accessible 24 hours a day to help and support. The fact that the sick person's condition varies from day to day affects the family members' control of their days. They manage this uncertain situation by spending all the time they can with the sick person, adapting to what the person's condition may be on a particular day. A consequence of their wish to be with the person as much as possible is that the family members neglect their own leisure pursuits and social activities. They are always ready to change their daily planning in accordance with how the person is feeling:

*We try to live as normal a life as possible, as close as we can to before. It is of course not the case during the periods when she is sick and does not have strength. Then we become very withdrawn, and we're not with people at all. We are at home, and I make all contacts outside and take care of everything that should be managed. (*Male, husband, age 60)

#### 5. Distracting oneself by being with others

The family members find it stressful to live close to a person with cancer and be continually reminded of how life has changed. They assume the next-of-kin role 24 hours a day and are always ready to offer comfort and do all they can. Sometimes they find it all so difficult that they feel a need to think thoughts other than those that habitually weigh on them. By being with other people and doing things with them, the family members manage to distract their thoughts. They can think of something else for a while and be themselves in the company of friends and co-workers. This provides emotional relief, a temporary diversion. In everyday life, the family members are weighed down with brooding over what the disease is going to involve. At the same time, they are continually reminded of their role as family members. In the company of others, they find temporary solace and escape from the lurking threat of death. For a while, they can get away from it all:

*There are periods when you just sort of think of something else and forget all the problems. I mean, when you meet friends you don't want them to talk about this. You want to leave it behind you at home. Otherwise it spoils the pleasure of the moment. *(Male, husband, age 60)

#### 6. Shielding the family from grief

The family members find it difficult to tell those closest to them about the disease and prognosis. They shield them by keeping back information and understating the gravity of the situation because they want the best for them. To spare them pain and suffering, the family members endure the burden alone. They want to keep their children and parents out of it and not put more of a burden on them than is necessary. In the case of children, the family members are uncertain as to how much information to disclose. They find it particularly difficult to be honest with children in this sort of situation. It would worry and upset the children to know that the person is going to die, and the family members want to spare them this. It is similar with their parents, whom they regard as having enough to do coping with their own situation and with aging. For this reason, the family members prefer to gloss over the seriousness of the situation:

*I don't want my family, my husband and children, to have to go through so much. I don't want it to affect them so much. So, I think there's no need for me to weigh them down with all that. *(Female, daughter, age 31)

#### 7. Attempting to maintain hope

Finding it unendurable to think about the threat of death hanging over the sick person, the family members do their utmost to think positive thoughts that suggest there is hope of a life together in the future. For the most part, the family members remain close to the person. They want to do all they can to keep up the person's spirits when dark thoughts make themselves felt. They keep the person company during treatment at the hospital. This treatment gives them hope, including the hope of other possible forms of treatment. The family members have both positive and negative thoughts about the situation, but try to concentrate on the positive ones. They can focus on hope, for instance, can instil it both in themselves and in the sick person and thus maintain a faith in the future, a *shared *future:

*I've tried to comfort him, of course. In the past people used to feel terrible from that sort of treatment but nowadays you can get a lot of help so that you aren't so sick. So you're hoping it'll be that way now as well. You've got to tell him the positive things and not mention the negative things you hear, as much as that's possible, and give him hope. So I suppose, all in all, I see the bright side more than the dark side when it comes down to it. I try to see things in a pretty positive light. *(Female, niece, age 48)

## Discussion

There are very few studies about being a family member of a person with lung or gastrointestinal cancer, two of the most common causes of death in Europe [17]. The findings in this study bring into focus the challenges confronting family members when one in the family has been diagnosed with advanced cancer. The family members live in a state of insecurity, unsure as to when indeed the frightening possibility will become reality. They cannot escape the feeling that this occasion is coming nearer with each day that passes. In the back of their minds, they prepare themselves for a frightening lonely life without their loved one, knowing that they are going to be left alone in grief but without the slightest idea *when*. Such fear of the future has been previously described in the literature [21,22].

In this study, the caregiving burden for family members was not particularly onerous in terms of time because the advanced cancer was in its early stage. Nevertheless, they had to handle life that changed after diagnosis and the overwhelming threat of death hanging over the sick person. How the family members in this study managed is summarized by the theme *Striving to be prepared for the painful*, which expresses the function of the seven underlying sub-themes or strategies. In a study performed by Persson and Sundin [3], the significant others were found to be 'Striving to function oneself' as best they could in everyday life whilst also attempting to visualize what the future held in store through 'Managing perceived threats'. This was confirmed by our own findings. In that same study, the significant others were found to be in an altered relationship with their next of kin, on the one hand closely attached to the sick person, and on the other hand more distanced with feelings of being in an unequal relationship [3]. The finding regarding an altered relationship with the sick person was not found in this study. These different findings may be explained by the fact that in the earlier study, the elapsed time after diagnosis was six months compared with about two months in our study. It is plausible that living longer with a person who has advanced cancer means larger inequality in the relationship because of an increased amount of caregiving by the family member.

The family members in our study had the strength to make things easier in everyday life. A similar strategy was reported previously and expressed as 'make the best of it' [22]. This means to not give up, be strong, look ahead, and do the things that can be done and solve practical problems. In Adelbratt and Strang's study [23], family members in the early stage of the illness were preparing practical things for the funeral and planning to travel with friends. A husband started to practice cooking so he could manage this task when his wife died. These previous findings, as well as the sub-theme in our study 'Making things easier in everyday life', relates to problem-focused coping. Problem-focused coping is concerned with handling the source of stress, dealing directly with the situation. This type of coping is more often related to successful outcomes [24] compared to emotion-focused coping. Emotion-focused coping is concerned with handling emotions associated with stressful situations, (i.e. relieving the feeling of stress without actually having to change the situation) [14,15]. However, longitudinal and experimental studies have documented the adaptive potential of emotional-approach coping in the context of several types of stressors, including breast cancer and chronic pain [25]. In our study, only 'Making things easier in everyday life' can be classified as problem-focused. The other sub-themes are emotion-focused.

The strategy 'Banishing thoughts about the approaching loss' revealed that family members experience the situation as highly emotionally stressful and have to manage it by distancing. This strategy is similar to findings in a previous study by Benkel and colleagues [26] indicating that family members fully understand that cancer is a serious and incurable disease that is leading to the patient's death. However, because the date of death is unknown, they hide this information deep in their hearts so they can live as if death is far off in the future. This makes it possible, despite the insight, to deal with the patient as a living person instead of a dying one. The strategy 'Thinking that the death is far off in the future' in this previous study as well as 'Banishing thoughts about the approaching loss' in our study are in contrast to the concept of denial, a psychological defence mechanism in which the person does not acknowledge the existing reality and suppresses thoughts of it [26].

'Living in the present' gives the family members a feeling that the catastrophe is ahead of them and not happening right now. They take one day at a time. Benkel and colleagues [26] showed that family members strive to maintain as normal a life as possible during the palliative stage, keeping to their usual routines and activities. In the findings of other studies, housekeeping, cooking and other practical activities were more prominent than in our study findings [26-28].

'Adjusting to the sick person's situation' includes the family members' sympathy for the sick person and their wish to be accessible every day to provide support. Milberg and Strang [29] found that family members do everything they can for the sick person in everyday life in palliative home care, supported by the sharing caring staff. Caring for the sick person with support from the staff gives the family member a feeling of greater togetherness and a deeper understanding of the sick person's everyday activities [29]. In our study, the family members' interview narratives showed that they wanted to be close to the sick person, spending all the time they could with their loved one. Goldzweig and colleagues [2009] reported that middle age and older males tend to have more support from females than vice versa [30]. In our study, nearly half of the family members were female and married to a male patient. The family members as well as patients were older adults. Perhaps the findings in our study were influenced by gender and age given that married older male patients have been shown to receive more support than married female patients.

'Distracting themselves by being with others' concerns the finding that friends and co-workers offer support to the family members, enabling them to be with others and think of something else for a short while [1,26,31]. Such support has been indicated in previous studies [32-34].

'Shielding the family and bearing their grief' means that the family members shield their family from the disease and poor prognosis. They think it best to protect the children from suffering by holding back information and understating the gravity of the situation. In a study by Fletcher and colleagues [35], family members were worried about their family's well-being, especially the children [35]. They felt pressured by the possibility that their children might face the same illness or that their children might need more emotional support than they were receiving. In addition, in our study the family member managed worries about their family by protecting the children from distress and bearing the grief themselves. The fact that there are not many studies showing that family members shield the others in the family and bear their grief can perhaps be explained by the fact that in recent years research has increasingly adopted the perspective of those who provide care rather than that of the close family.

'Setting hopes on the future' was the strategy least frequently mentioned by the family members in the interviews. One possible explanation for this is that the cancer diagnosis was received 2-3 months ago, and some family members could have still been in shock or not yet adjusted to the new reality of the person's having advanced cancer. Previous research on hope has usually been directed towards the terminal stage of cancer. It has focused mainly on the significance of hope from the patient's perspective as a way to manage terminal illness through acknowledgement, acceptance and struggle. There are two overarching themes of hope commonly described in the literature: 'living with hope' (reconciliation with life and death) and 'hoping for something' (hope of being cured) [36,37]. The findings in the present study indicate that the family members were indeed 'hoping for something'. This is a reasonable result in view of the fact that all of the sick persons were undergoing cytostatic treatment. The few studies concerning hope in family members show inconsistent findings regarding the influence of age on the existence of hope [36-38]. Prognostic uncertainty and the continued hope of survival harboured by family members can make it difficult to decide the proper time to stop active therapy and initiate palliation [37].

### Methods discussion

The findings in this study, for instance, that family members mostly give emotional support to the sick person reflect the early stage of advanced cancer. This must be borne in mind when interpreting these findings, especially in view of the fact that previous studies have, with few exceptions, concerned a later stage of palliative or terminal care. One weakness with regard to the transferability of the findings is the small proportion of male participants (20%). The nurses who inquired about participation were both males and females. However, 13 of the 20 patients were male, and all of them except one chose a female family member. Four of the 7 female patients were mothers, and 3 of them chose a daughter. The design of the study stipulated that the family member be chosen by the sick person, which might be one reason for the low proportion of men among family members. This needs to be kept in mind when comparing the findings in this study with findings in previous research.

The achievement of credibility in the inductive approach for qualitative content analysis implies careful and comprehensive interpretation at every stage of the research process [20]. This includes an appraisal of the authors regarding whether the data reach enough saturation and credibility. The family members appreciated being able to talk about their situation, and the interviews were characterized by richness of narrations. The duration of the interviews was on average 75 minutes. However, some of the family members were preoccupied with their experienced distress and had difficulty finding words for how they managed the situation. In these cases, several follow-up questions were needed to obtain comprehensive descriptions. The design applied followed the basic principles of latent content analysis, which means that there was a systematic coding into sub-themes and then integration into a theme [20]. The construction of the concepts was derived from the in-depth analysis and constant comparison of parts within the perspective of an emerging wholeness. To attain greater rigour, the analysis by the first author (CS) was scrutinized at each step by the other two authors (GA, BH), both of whom are experienced in latent content analysis. The findings were discussed until agreement was reached.

## Conclusions

The function of the management strategies in this study was *Striving to be prepared for the painful*. Family members try to make things easier in everyday life, both for the sick person and for themselves, and they visualize their future life being left alone as a single parent or without their partner. They do what they can to make the most of the here and now for all involved, trying as it were, to carry the whole family in their arms.

Much previous research has focused on distress in relation to advanced cancer. The results in this study contribute new knowledge about management strategies of family members of a person in the early stage of advanced lung or gastrointestinal cancer. When constructing psychosocial intervention programmes, it is very important for health care professionals to have knowledge about how family members handle this stressful life situation successfully. A psychosocial programme needs to focus on prevention based on the anticipated increased caregiving burden, which may represent a significant risk of ill-health in family members.

Even though the findings indicate that the family members' own resources were sufficient in enabling them to handle the difficult situation in an acceptable way, it is important to generate knowledge from a longitudinal perspective of the whole cancer trajectory experienced by a family member of a person with incurable lung or gastrointestinal cancer. More knowledge about family members' management strategies, well-being and physical health is needed before designing evidence-based intervention programmes that prevent ill-health in the family members, including identifying those who are most vulnerable. Future research is also needed to investigate whether family members' management strategies differ based on diagnosis, relationship, age or gender and whether certain coping strategies are generic.

## Competing interests

The authors declare that they have no competing interests.

## Authors' contributions

CS and GA designed the study. CS conducted the interviews and made the initial analysis of the interview transcripts. Each step of the analysis was then scrutinized and discussed by all three authors, and all the authors read and approved the final manuscript.

## Pre-publication history

The pre-publication history for this paper can be accessed here:

http://www.biomedcentral.com/1472-6955/10/18/prepub
